# Effect of vaccine hesitancy on female college students’ willingness to receive the HPV vaccine in China: a multicenter cross-sectional study

**DOI:** 10.1186/s12889-024-19303-1

**Published:** 2024-07-18

**Authors:** Xiaoxue Li, Fengzhi Zhang, Manman Li, Chunhui Lin, Kaige Shi, Fangfang Yang

**Affiliations:** Department of Gynecology, The Third Affiliated Hospital of Zhengzhou University, 7 Front Kangfu Street, Zhengzhou, Henan province 450052 China

**Keywords:** Vaccine hesitancy, HPV vaccine, Vaccination intention, Influencing factors, College students

## Abstract

**Objective:**

To analyse the influencing factors of vaccine hesitancy on HPV vaccination willingness of female college students in order to promote the promotion of HPV vaccine in female college student population.

**Methods:**

From September-October 2022, a convenience sampling method was used to conduct a questionnaire survey among freshman female students from four higher vocational colleges in Henan Province, China. The survey comprised a general information questionnaire, as well as inquiries regarding vaccine hesitancy and willingness to receive the human papillomavirus (HPV) vaccine. In PSM analyses, vaccine-hesitant students were matched with non-vaccine-hesitant students at a 1:1 ratio; subsequently, both univariate and multivariatble logistic regression analyses were applied to assess the impact of vaccine hesitancy on female university students’ willingness to receive the HPV vaccine.

**Results:**

The results revealed a vaccine hesitancy rate of 44.75% among female university students, with 82.9% expressing willingness to receive the HPV vaccine. The results of the multivariable ordinal logistic regression analysis indicated vaccine hesitancy is a risk factor for HPV vaccination intentions among female university students [*OR* = 4.38, 95% CI (2.74, 6.99), *P* < 0.001]. Furthermore, the field of study (*P* = 0.01) and independently seeking information about the HPV vaccine (*P* = 0.04) were identified as factors influencing female university students’ willingness to receive the HPV vaccine.

**Conclusions:**

Non-vaccine-hesitant students were more likely to be willing to receive the HPV vaccine than vaccine-hesitant students. Healthcare providers and educators should focus on vaccine attitudes among female college students to reduce vaccine hesitancy and enhance community education on cervical cancer, HPV infection and prevention through multichannel campaigns.

## Introduction

Cervical cancer is the fourth most common malignant tumour threatening the health of women worldwide. According to Global Cancer Statistics 2021 [1], approximately 600,000 new cases of cervical cancer are reported globally each year, with approximately 340,000 of those cases ending in death. In China, the yearly incidence of cervical cancer is approximately 131,000 cases, with a death toll of approximately 53,000, accounting for 18.4% of all female malignant tumour-related deaths [2]. Astoundingly, high-risk human papillomavirus (HPV) infection accounts for 99% of cervical cancer cases. Persistent HPV infections can cause cervical cancer in women, even though the majority of infections tend to naturally progress without producing any symptoms [3]. HPV vaccination can lower the incidence of cervical cancer by approximately 70%, in addition to preventing other diseases, such as genital warts, oropharyngeal cancer, and anal cancer [4]. The rates of HPV vaccination coverage among female university students in Australia [5], Portugal, and the United Kingdom [6] exceed 80%. In Hong Kong, China, the vaccination rate among university students is 47.20% [7]. Zhang Xiaoxiao et al. [8] conducted a survey of 3007 female university students in four provinces of China using multistage sampling and convenience sampling and showed that the HPV vaccination rate was 2.96%, similar to the results of the Tianjin study [9]. It is evident that the HPV vaccination rate among Chinese female university students remains comparatively low.

Research indicates [10] that premarital sexual activity is becoming increasingly common among Chinese university students, leading to an increased risk of HPV infection. In China, the first peak of high-risk HPV infection occurs between the ages of 15 and 24 years in females [11]. In addition to being in the age group most at risk of contracting HPV, female university students are also people of reproductive age. Female university students’ views affect the HPV vaccination rates of the upcoming generation of young women as well as the current vaccination rates [12]. Furthermore, model studies [13, 14] have demonstrated that combining HPV vaccination with cervical cancer screening for eligible women is a highly cost-effective preventive measure in China. Hence, encouraging female university students to be vaccinated against HPV is essential for preventing cervical cancer and other diseases linked to HPV.

Vaccine hesitancy was identified as one of the top ten global health hazards in 2019 by the World Health Organization (WHO) [15]. Vaccine hesitancy is defined as the decision to forgo or postpone vaccination even when immunization services are available. Although HPV vaccination can significantly reduce the incidence of high-risk HPV infection, vaccine hesitancy remains pervasive worldwide. Based on the 3 C model [16, 17], vaccine hesitation can be influenced by various factors such as vaccine pricing [15], affordability [18], geographic accessibility [16], faith in healthcare experts to urge vaccination [16, 19, 20], vaccine efficacy and safety [20], and lack of perceived threat of HPV-related diseases [21]. According to reports, HPV vaccination rates have decreased in many countries and regions due to HPV vaccine hesitancy. For example, in 2013, HPV vaccine hesitancy became widespread in Japan after the media reported that the administration of untested vaccines could lead to adverse events, resulting in a significant decline in HPV vaccination rates throughout the country [22]. A study conducted among medical students in Brazil revealed that vaccine hesitancy is a primary determinant of low HPV vaccination rates [23]. Uncertainty regarding the safety of the HPV vaccine has emerged as the leading cause of vaccine hesitancy among medical students [24]. At present, vaccine hesitancy poses a substantial obstacle to the widespread adoption of HPV vaccination among the target age group. While numerous studies have been conducted to explore vaccine hesitancy and attitudes towards vaccination both domestically and internationally [25–27], confounding factors, such as demographic characteristics, have not been effectively controlled for, thereby compromising the accuracy of the research findings.

## Objectives and methods

### Study design and population

The present study involved a cross-sectional survey conducted using a stratified, cluster sampling method. From September to October 2022, convenience sampling was employed to select female first-year college students aged 18 years and older from four higher educational institutions in Henan Province, consisting of two medical colleges and two nonmedical colleges, totalling eight classes. All participating students were informed of the research objectives and were required to provide online informed consent forms prior to enrolment. The electronic questionnaires were distributed through the QuestionStar platform. In cases where the participating students left unanswered questions, prompts were sent via the QuestionStar platform. Research participants were selected based on the inclusion and exclusion criteria.

### Study population

#### Inclusion criteria

The inclusion criteria were as follows: ① aged ≥ 18 years; ② first-year female college students; ③ students with the self-reported absence of contraindications to vaccine administration; and ④ students who volunteered to participate and provided informed consent.

The exclusion criteria were as follows: ① severe organ disease, such as coronary heart disease, chronic renal failure, or similar conditions; and ② previous participation in similar studies.

The dropout criterion was voluntary withdrawal for personal reasons.

#### Sample size

To estimate the sample size, the following formula was utilized: $n=\frac{{Z}_{1-\alpha /2}^{2}\times P\left(1-P\right)}{{\delta }^{2}}(\alpha =0.05,{Z}_{1-\alpha /2}=1.96)$. In this study, the number of female college students surveyed (n) and their expected willingness to receive the HPV vaccine (p) were determined. Recent meta-analyses [28, 29] conducted in China indicated that 68.0–71.8% of Chinese female college students were willing to receive the HPV vaccine. Therefore, 688 female college students would be needed for the survey. Considering the study’s uncontrolled elements and the use of PSM, the sample size was suitably increased to 1043 participants.

### Research tools

#### General information survey

Regarding factors associated with vaccine hesitancy and HPV vaccination willingness among female college students, based on a literature review [30, 31], the following 17 factors were considered in this study: age, field of study, smoking habit, parental/maternal education level, average monthly household income, romantic relationship status, sexual activity history, affordability of the HPV vaccine for parents, affordability of the HPV vaccine for oneself, vaccination status of individuals in the surrounding community, self-obtained HPV vaccine information, presence of healthcare professionals in the family, acceptability of vaccine price, presence of cancer cases among relatives or friends, and previous receipt of other self-funded vaccines.

#### Vaccine hesitancy scale

The Adult Vaccine Hesitancy Scale (AVHS) is commonly utilized to measure vaccine hesitancy among adult populations [32]. The AVHS was developed by Peretti-Watel et al. in 2015 as an extension of the Vaccine Hesitancy Scale [33]. In studies examining adult attitudes and hesitancy towards coronavirus disease 2019 (COVID-19) vaccination in the United States, Poland [34], Italy [35], and other countries [36], the AVHS had a Cronbach’s α coefficient of 0.893 (USA), indicating strong validity and reliability. Lu [34] verified the validity and reliability of the Chinese version of the questionnaire, reporting a Cronbach’s α coefficient of 0.729. The AVHS contains ten items and two dimensions: three items from the Risk dimension (Items 5, 9, and 10) and seven items from the Trust Lacking dimension (Items 1, 2, 3, 4, 6, 7, and 8). A 5-point Likert scale, with values ranging from 1 to 5 (strongly agree to strongly disagree), was used by participants to indicate their agreement. Items 5, 9, and 10 are reverse scored, indicating that a higher score reflects a stronger level of vaccine hesitancy. Furthermore, the AVHS has been used to measure hesitancy levels towards influenza vaccination [32] and herpes zoster vaccination, among others [34]. The scale’s Cronbach’s α coefficient in the present investigation was 0.705. Using a cut-off value of 25 points, study participants with AVHS scores ≥ 25 points were classified into the vaccine-hesitant group, while those with scores < 25 points were classified into the non-vaccine-hesitant group.

#### Willingness to receive the HPV vaccine

An inquiry was conducted among female university students to assess their willingness to receive the HPV vaccine. The question posed was “Are you willing to receive the HPV vaccine?” The response options included “willing,” “neutral/unsure,” and “unwilling.”

### Data collection

**Fig. 1:**
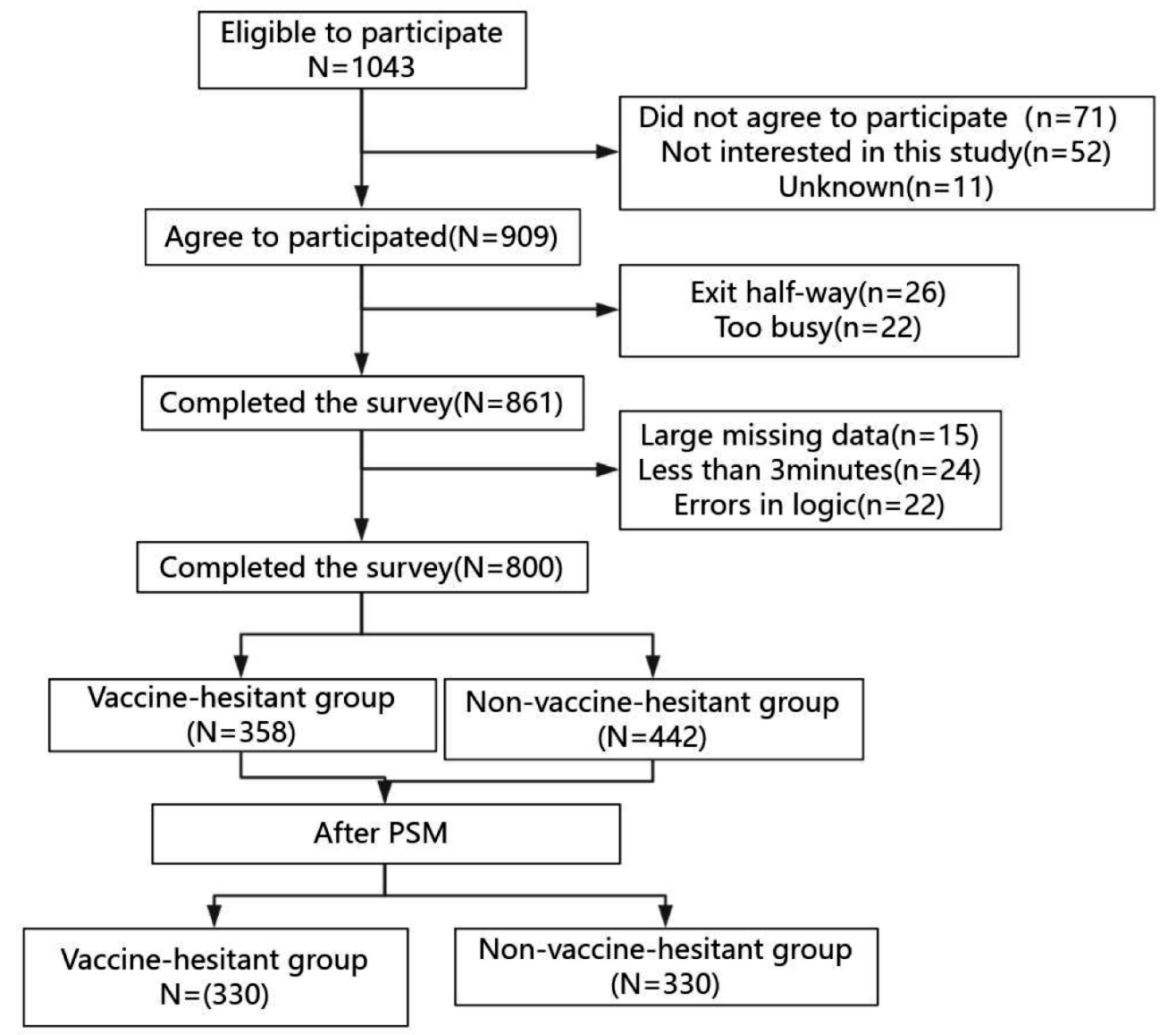
A flow chart of the participants

The survey was conducted using the online platform QuestionStar. This platform automatically generates QR codes or URL links for manually inputted survey questionnaires. Participants were then prompted to fill out the questionnaire within 15 min by scanning the QR code or clicking the link. In the case of unanswered questions, QuestionStar provided reminders. Additionally, all collected data was manually scrutinized to identify questionnaire completion times (excluding surveys completed in under 3 min) and to ensure logical and reasonable answers (e.g., eliminating surveys with identical selected options). The extracted data were cross-checked by two members of the research team to ensure accuracy and further organize the data. In this study, HPV vaccination willingness served as the outcome variable, while vaccine hesitancy scores were utilized as the grouping variable, distinguishing between the vaccine-hesitant and non-vaccine-hesitant groups (see Fig. 1).

### Statistical analysis

Data analysis was performed using Stata 16.0 and SPSS 25.0 software. Stata was utilized for PSM, and SPSS was used for the remaining analyses. Categorical data are presented as frequencies and percentages, and between-group comparisons were conducted using chi-square tests. Continuous data are presented as means, standard deviations, medians, or quartiles, and between-group comparisons were performed using independent t tests or rank sum tests. Two-sided *P* < 0.05 was considered to indicate a statistically significant difference. PSM, a statistical method for controlling confounding factors, was applied in this study. This study used HPV vaccine acceptance as the outcome variable and vaccine hesitancy as the grouping variable. There were 17 potential confounding factors related to vaccine hesitancy and HPV vaccine acceptance, which were treated as covariates. The nearest neighbour matching method was employed for PSM with a 1:1 matching ratio and a calliper value of 0.02. The balance of covariates between the non-vaccine-hesitant and vaccine-hesitant groups was tested after matching. Willingness to receive the HPV vaccine was used as the dependent variable for univariate and multivariate analyses. For univariate analysis, single-factor multivariable ordinal logistic regression analysis was performed, while for multivariate analysis, multivariable ordinal logistic regression analysis was utilized. A significance level of α = 0.05 (two-tailed) was used for the tests.

## Results

### General information of the study participants

The study included a total of 861 participants, with a response rate of 92.9%. The data of 800 female university students were collected for this study, with a response rate of 92.9%; 358 students (44.75%) were included in the vaccine-hesitant group, and 442 students (55.25%) were in the non-vaccine-hesitant group. The average age was 18.56 ± 0.741 years.

### General characteristics of the two student groups before PSM

Before PSM, there was a statistically significant difference between the two groups in terms of the age distribution (*P* = 0.001), vaccination status of individuals in the students’ social circles (*P* = 0.002), and presence of cancer cases among relatives or friends (*P* = 0.006). Refer to Table 1.

**Table 1:** Comparison of the general information of the students in the two groups before propensity score matching

Variables	Non-vaccine-hesitant group	Vaccine-hesitant group	Statistical value	*P* value
	(*n* = 442)	(*n* = 358)		
Age	18.52 ± 0.664	18.62 ± 0.824	11.984^1)^	0.001
Field of study			0.003^2)^	0.959
Nursing	235(53.20)	191(53.40)		
Non-nursing	207(46.80)	167(46.60)		
Smoking habit			0.158^2)^	0.924
Ever	13(2.90)	9(2.50)		
Never	421(95.20)	343(95.80)		
Current	8(1.80)	6(1.70)		
Paternal education level			-1.155^3)^	0.248
Junior high school or below	323(73.10)	274(76.50)		
High school or junior college	90(20.40)	66(18.40)		
Professional training college	23(5.20)	12(3.40)		
Undergraduate	6(1.40)	6(1.70)		
Maternal education level			-1.003^3)^	0.316
Junior high school or below	349(79.00)	292(81.60)		
High school or junior college	67(15.20)	53(14.80)		
Professional training college	20(4.50)	6(1.70)		
Undergraduate	6(1.40)	7(2.00)		
Average monthly household income			-0.457^3)^	0.648
≤ 3000	164(37.10)	121(33.80)		
3000–6000	210(47.50)	187(52.20)		
6001–9000	51(11.50)	42(11.70)		
≥ 9000	17(3.80)	8(2.20)		
Relationship status			1.966^2)^	0.374
Not currently	254(57.50)	204(57.00)		
Ongoing	75(17.00)	73(20.40)		
Have ever	113(25.60)	81(22.60)		
Residence			0.557^2)^	0.455
Countryside	354(80.10)	279(77.90)		
City	88(19.90)	79(22.10)		
Sexual activity history			1.909^2)^	0.385
Yes	19(4.30)	9(2.50)		
No	396(89.60)	328(91.60)		
Don’t want to talk about it	27(6.10)	21(5.90)		
Affordability of the HPV vaccine for parents			0.439^2)^	0.507
Yes	198(44.80)	152(42.50)		
No	244(55.20)	206(57.50)		
Affordability of the HPV vaccine for oneself			0.657^2)^	0.418
Yes	35(7.90)	23(6.40)		
No	407(92.10)	335(93.60)		
Has anyone in your social circle received the HPV vaccine?			9.593^2)^	0.002
Yes	141(31.90)	79(22.10)		
No	301(68.10)	279(77.90)		
Self-acquisition of HPV vaccine-related knowledge			0.809^2)^	0.369
Yes	267(60.40)	205(57.30)		
No	175(39.60)	153(42.70)		
Is there a healthcare worker in your family?			0.005^2)^	0.945
Yes	60(13.60)	48(13.40)		
No	382(86.40)	310(86.60)		
Acceptable HPV vaccine prices RMB			-0.502^3)^	0.615
≤ 500	274(62.00)	222(62.00)		
500–1000	107(24.20)	104(29.10)		
1000–2000	52(11.80)	25(7.00)		
≥ 2000	9(2.00)	7(2.00)		
Presence of cancer cases among relatives or friends			7.485^2)^	0.006
Yes	137(31.00)	80(22.30)		
No	305(69.00)	278(77.70)		
Have you received any other privately funded vaccines?			0.754^2)^	0.385
Yes	156(35.30)	137(38.30)		
No	286(64.70)	221(61.70)		

*Note*^1)^ indicates an independent sample t test; ^2)^ indicates a chi-square test; ^3)^ indicates a rank-sum test

### General characteristics of the two student groups after PSM

After PSM, a total of 330 students were paired, leading to a convergence of all factors, devoid of statistically significant differences (*P* > 0.05). Refer to Table 2 for detailed information.

**Table 2:** Comparison of the general information of the students in the two groups after propensity score matching

Variables	Non-vaccine-hesitant group	Vaccine-hesitant group	Statistical value	*P* value
	(*n* = 330)	(*n* = 330)		
Age	18.57 ± 0.704	18.54 ± 0.756	0.654^1)^	0.48
Field of study			0.006^2)^	0.938
Nursing	181(54.80)	180(54.50)		
Non-Nursing	149(45.20)	150(45.50)		
Smoking habit			0.526^2)^	0.769
Ever	12(3.60)	9(2.70)		
Never	313(94.80)	315(95.50)		
Current	5(1.50)	6(1.80)		
Paternal education level			-0.210^3)^	0.834
Junior high school or below	250(75.80)	248(75.20)		
High school or junior college	63(19.10)	64(19.40)		
Professional training college	17(5.20)	18(5.50)		
Maternal education level			-0.510^3)^	0.610
Junior high school or below	271(82.10)	265(80.30)		
High school or junior college	42(12.70)	52(15.80)		
Professional training college	12(3.60)	6(1.80)		
Undergraduate	5(1.50)	7(2.10)		
Average monthly household income			-0.830^3)^	0.407
≤ 3000	126(38.20)	111(33.60)		
3000–6000	156(47.30)	173(52.40)		
6001–9000	38(11.50)	39(11.80)		
≥ 9000	10(3.00)	7(2.10)		
Relationship status			0.943^2)^	0.624
Not currently	186(56.40)	184(55.80)		
Ongoing	60(18.20)	69(20.90)		
Have ever	84(25.50)	77(23.30)		
Residence			0.863^2)^	0.649
Countryside	265(80.30)	262(79.40)		
City	65(19.70)	68(20.60)		
Sexual activity history			0.085^2)^	0.771
Yes	11(3.30)	9(2.70)		
No	295(89.40)	302(91.50)		
Don’t want to talk about it.	24(7.30)	19(5.80)		
Affordability of the HPV vaccine for parents			0.503^2)^	0.478
Yes	135(40.90)	144(43.60)		
No	195(59.10)	186(56.40)		
Affordability of the HPV vaccine for oneself			0.023^2)^	0.88
Yes	24(7.30)	23(7.00)		
No	306(92.70)	307(93.00)		
Has anyone in your social circle received the HPV vaccine?			0.008^2)^	0.927
Yes	78(23.60)	79(23.90)		
No	252(76.40)	251(76.10)		
Self-acquisition of HPV vaccine-related knowledge			0.100^2)^	0.752
Yes	195(59.10)	191(57.90)		
No	135(40.90)	139(42.10)		
Is there a healthcare worker in your family?			0.214^2)^	0.644
Yes	45(13.60)	41(12.40)		
No	285(86.40)	289(87.60)		
Acceptable HPV vaccine prices RMB			-0.714^3)^	0.475
≤ 500	214(64.80)	201(60.90)		
500–1000	76(23.00)	97(29.40)		
1000–2000	36(10.90)	25(7.60)		
≥ 2000	4(1.20)	7(2.10)		
Presence of cancer cases among relatives or friends			0.034^2)^	0.854
Yes	76(23.00)	78(23.60)		
No	254(77.00)	252(76.40)		
Have you received any other privately funded vaccines?			0.026^2)^	0.871
Yes	116(35.20)	118(35.80)		
No	214(64.80)	212(64.20)		

*Note*^1)^ indicates an independent sample t test; ^2)^ indicates a Chi-square test; ^3)^ indicates a rank-sum test

### Univariate analysis of willingness to receive the HPV vaccine

A univariate analysis of female university students’ willingness to receive the HPV vaccine was conducted before and after matching. Prior to PSM analysis, the proportional odds assumption test was performed on each variable to test the proportional odds assumption for the ordered logistic regression. The analysis revealed statistically significant differences in female university students’ willingness to receive the HPV vaccine when comparing different fields of study (*P* = 0.001), the affordability of the HPV vaccine for parents (*P* = 0.021), the presence of individuals in the surrounding community who had been vaccinated against HPV (*P* < 0.001), the self-acquisition of HPV vaccine-related knowledge (*P* = 0.007), and the presence of vaccine hesitancy (*P* < 0.001). Other differences were not statistically significant (*P* > 0.05).

Following PSM analysis, the proportional odds assumption test was conducted on each variable to satisfy the test of proportional odds assumption for the ordered logistic regression. The analysis revealed statistically significant differences in female university students’ willingness to receive the HPV vaccine when comparing different fields of study (*P* = 0.005), self-acquisition of HPV vaccine-related knowledge (*P* = 0.014), and the presence of vaccine hesitancy (*P* < 0.001). Other differences were not statistically significant (*P* > 0.05). Refer to Tables 3 and 4.

**Table 3:** Univariate and multivariate analyses of female university students’ willingness to receive the HPV vaccine before PSM

Variables	Univariate analysis		Multivariate analysis	
	OR(95% CI)	*P* value	OR(95% CI)	*P* value
Age	0.74(0.59–0.93)	0.011	0.80(0.63–1.02)	0.074
Field of study				
Nursing	1.89(1.24–2.75)	0.001	1.84(1.24–2.75)	0.003
Non-Nursing				
Smoking habit				
Ever	0.54(0.09–3.28)	0.51		
Never	0.80(0.18–3.64)	0.77		
Current				
Paternal education level				
Junior high school or below	1.26(0.60–2.65)	0.54		
High school or junior college	1.11(0.50–2.53)	0.8		
Professional training college				
Undergraduate				
Maternal education level				
Junior high school or below	1.59(0.44–5.70)	0.48		
High school or junior college	1.53(0.40–5.90)	0.53		
Professional training college	1.32(0.27–6.48)	0.73		
Undergraduate				
Average monthly household income				
≤ 3000	0.44(0.10–1.96)	0.28		
3000–6000	0.39(0.09–1.71)	0.21		
6001–9000	0.38(0.08–1.79)	0.22		
≥ 9000				
Relationship status				
Not currently	0.59(0.36–0.95)	0.029	0.65(0.39–1.08)	0.093
Ongoing	0.94(0.50–1.76)	0.839	0.97(0.50–1.88)	0.923
Have ever				
Residence				
Countryside	1.13(0.73–1.76)	0.585		
City				
Sexual activity history				
Yes	6.27(0.75–52.46)	0.091		
No	1.09(0.51–2.30)	0.828		
Don’t want to talk about it.				
Affordability of the HPV vaccine for parents				
Yes	1.57(1.07–2.30)	0.021	1.44(0.95–2.18)	0.084
No				
Affordability of the HPV vaccine for oneself				
Yes	0.80(0.41–1.56)	0.511		
No				
Presence of individuals in the surrounding community who have been vaccinated against HPV				
Yes	2.71(1.63–4.53)	0	2.00(1.16–3.45)	0.012
No				
Self-acquisition of HPV vaccine-related knowledge				
Yes	1.66(1.15–2.40)	0.007	1.43(0.96–2.12)	0.078
No				
Is there a healthcare worker in your family?				
Yes	1.60(0.87–2.96)	0.13		
No				
Acceptable HPV vaccine prices RMB				
≤ 500	0.99(0.28–3.58)	0.99		
500–1000	1.05(0.28–3.88)	0.95		
1000–2000	3.27(0.69–15.47)	0.14		
≥ 2000				
Presence of cancer cases among relatives or friends				
Yes	1.40(0.90–2.17)	0.132		
No				
Have you received any other privately funded vaccines?				
Yes	1.33(0.90–1.98)	0.151		
No				
Vaccine Hesitancy				
Non-vaccine-hesitant group	5.13(3.37–7.82)	<0.001	4.98(3.23–7.68)	<0.001
			-2 log-likelihood value	388.736
			Cox and Snell	0.123
			Nagelkerke	0.192
			McFadden	0.128
			Proportional odds assumption test	*P* = 0.826

**Table 4:** Univariate and multivariate analyses of female university students’ willingness to receive the HPV vaccine after PSM

Variables	Univariate analysis		Multivariate analysis	
	OR(95% CI)	*P* value	OR(95% CI)	*P* value
Age	0.95(0.73–1.25)	0.72		
Field of study				
Nursing	1.78(1.19–2.66)	0.005	1.73(1.14–2.64)	0.01
Non-Nursing				
Smoking habit				
Ever	0.676(0.11–4.22)	0.675		
Never	1.02(0.22–4.84)	0.977		
Current				
Paternal education level				
Junior high school or below	1.90(0.87–4.14)	0.105		
High school or junior college	1.48(0.63–3.49)	0.37		
Professional training college				
Undergraduate				
Maternal education level				
Junior high school or below	1.73(0.48–6.35)	0.404		
High school or junior college	1.54(0.39–6.09)	0.541		
Professional training college	1.21(0.22–6.49)	0.826		
Undergraduate				
Average monthly household income				
≤ 3000	0.65(0.14–2.95)	0.57		
3000–6000	0.58(0.13–2.62)	0.48		
6001–9000	0.59(0.12–2.88)	0.51		
≥ 9000				
Relationship status				
Not currently	0.66(0.39–1.09)	0.11		
Ongoing	0.95(0.50–1.84)	0.87		
Have ever				
Residence				
Countryside	1.24(0.77-2.00)	0.38		
City				
Sexual activity history				
Yes	3.74(0.43–32.79)	0.23		
No	0.88(038-2.02)	0.76		
Don’t want to talk about it.				
Affordability of the HPV vaccine for parents				
Yes	1.40(0.92–2.11)	0.12		
No				
Affordability of the HPV vaccine for oneself				
Yes	0.71(0.35–1.44)	0.34		
No				
Presence of individuals in the surrounding community who have been vaccinated against HPV				
Yes	1.77(1.04-3.00)	0.03	1.07(0.97–2.90)	0.07
No				
Self-acquisition of HPV vaccine-related knowledge				
Yes	1.65(1.11–2.47)	0.014	1.54(1.01–2.35)	0.04
No				
Is there a healthcare worker in your family?				
Yes	1.74(0.87–3.47)	0.12		
No				
Acceptable HPV vaccine prices RMB				
≤ 500	0.96(0.20–4.57)	0.96		
500–1000	0.90(0.19–4.41)	0.90		
1000–2000	2.45(0.41–14.75)	0.33		
≥ 2000				
Presence of cancer cases among relatives or friends				
Yes	1.08(0.67–1.73)	0.765		
No				
Have you received any other privately funded vaccines?				
Yes	1.50(0.97–2.32)	0.07		
Vaccine Hesitation				
Non-vaccine-hesitant group	4.25(2.68–6.75)	0	4.38(2.74–6.99)	0
			-2 log-likelihood value	86.747
			cox and Snell)	0.088
			Nagelkerke)	0.135
			(McFadden)	0.088
			Proportional odds assumption test	*P* = 0.381

### Multivariate ordered logistic regression analysis of factors influencing willingness to receive the HPV vaccine

Multivariate ordered logistic regression analyses were performed before and after matching, using HPV vaccination willingness (0 = unwilling, 1 = neutral/undecided, 2 = willing) as the response variable. Significant factors identified in the univariate analysis were used as explanatory variables in the multivariate analysis.

Prior to PSM, the proportional odds assumption test was conducted on the variables (χ2 = 4.331, *P* = 0.826), which indicated compliance with the proportional odds assumption test for multivariate ordered logistic regression. The results of the multivariate analysis (Table 3) demonstrated that field of study [*OR* = 1.84, 95% *CI* (1.24, 2.75), *P* = 0.003 ], the presence of individuals in the surrounding community who had been vaccinated against HPV [*OR* = 2.00, 95% *CI* (1.16, 3.45), *P* = 0.012 ], and vaccine hesitancy [OR = 4.98, 95% CI (3.23, 7.65), *P* < 0.001] were factors influencing HPV vaccine uptake among female university students.

After PSM, the proportional odds assumption test was conducted on the variables (*χ2* = 4.191, *P* = 0.381), which demonstrated adherence to the proportional odds assumption test for multivariate ordered logistic regression. The results of the multivariate analysis (Table 4) revealed that field of study [*OR* = 1.73, *95% CI* (1.14, 2.64), *P* = 0.01 ], self-acquisition of HPV vaccine-related knowledge [*OR* = 1.54, *95% CI* (1.01, 2.35), *P* = 0.04 ], and vaccine hesitancy [*OR* = 4.38, *95% CI* (2.74, 6.99), *P* < 0.001] were factors influencing HPV vaccination willingness among female university students.

## Discussion

### Scientific validity of evaluating students using propensity score matching

By utilizing PSM, a portion of the data was discarded in this study, which not only minimally impacted the statistical power but also effectively controlled for confounders [37]. Before PSM, there were statistically significant differences observed between non-vaccine-hesitant students and vaccine-hesitant students in terms of age (*P* = 0.001), whether their peers had received the HPV vaccine (*P* = 0.002), and whether their relatives or friends had experienced cancer (*P* = 0.006). However, after matching, the two groups achieved covariate balance, with all factors noticeably aligning. PSM ensured the accuracy of the impact of vaccine hesitancy on female college students’ willingness to receive the HPV vaccine in this study.

### Factors influencing female university students’ willingness to receive the HPV vaccine

#### Vaccine hesitancy

The results of this study indicated that vaccine hesitancy could lead to a decrease in willingness to receive the HPV vaccine among female university students [*OR* = 4.38, 95% *CI* (2.74, 6.99), *P* < 0.001], which aligns with previous research conclusions [15, 25–27], highlighting the association between vaccine hesitancy and reduced willingness to receive the HPV vaccine. Vaccine hesitancy can diminish the readiness of female college students to receive the HPV vaccine, and potential reasons for this phenomenon include limited awareness and insufficient trust in the HPV vaccine due to its relatively recent introduction in China compared to other vaccines. Additionally, public concerns regarding vaccine efficacy and safety, sparked by recent vaccine-related incidents [38], might contribute to vaccine hesitancy. Despite the recognition of the HPV vaccine as the sole anticancer vaccine, some students perceive it as unsafe and unnecessary. A lack of confidence in the vaccine’s effectiveness is the most frequent justification for refusing vaccination [39]. Providing accurate information about the importance, safety, and efficacy of HPV vaccination in preventing cervical cancer can significantly improve attitudes towards the HPV vaccine [40]. Previous studies [41, 42] have also demonstrated that confidence is the most influential factor in vaccine hesitancy, and safety and efficacy are the two crucial considerations in determining vaccine acceptance. University students who receive recommendations from and trust healthcare professionals are less likely to be hesitant to receive the HPV vaccine, as healthcare providers serve as the primary source of crucial information for the majority of this population (80.4%) [15]. Therefore, healthcare professionals can play a vital role in disseminating relevant knowledge and providing recommendations during diagnosis and other encounters, aiding in accurate comprehension of the risks of cervical cancer and the benefits of HPV vaccination [43].

Numerous experts have also proposed strategies to address vaccine hesitancy, encompassing the dissemination of information regarding disease sensitivity and severity, as well as vaccine effectiveness and safety [44, 45]. Larson, in particular, advocated for an emphasis on government attentiveness to public concerns and comprehension of public perspectives. Additionally, she suggested the publication of vaccine market authorization procedures and postmarket surveillance data, thereby enhancing transparency beyond mere considerations of vaccination program decisions [46, 47].

#### The influence of major and self-acquired knowledge of HPV vaccination on the female university students’ willingness to receive the HPV vaccine was examined in this study

Compared with nonmedical students, nursing students were more willing to receive the HPV vaccine [*OR* = 1.73, 95% *CI* (1.14, 2.64), *P* = 0.01]. Medical students, in particular, often exhibited higher levels of cognition [48], demonstrating a better understanding of the pathogenesis of HPV and the safety and effectiveness of the HPV vaccine. Additionally, medical students tended to place greater emphasis on their own physical health than did students in other disciplines [49], as they displayed greater disease prevention awareness. Consequently, the acceptance of the HPV vaccine was greater among nursing students.

Female university students who independently acquired HPV vaccine knowledge were more willing to receive the vaccine [*OR* = 1.54, 95% *CI* (1.01, 2.35), *P* = 0.04]. This trend can be attributed to the close association between the HPV vaccine and cervical cancer prevention. Students who actively sought out and comprehended HPV vaccine information often exhibited higher levels of vaccine literacy [50, 51], leading to increased vaccine acceptance.

The limitations of this research are as follows. First, the online surveys may be subject to selection bias because they only targeted university students interested in the HPV vaccine. Second, this study employed convenience sampling, which may introduce sampling errors. Third, a self-administered questionnaire was used in this study; the study participants were highly subjective when self-reporting, and the results of the study may be subject to information bias. Fourth, since this study included only students from four universities in the Chinese province of Henan, its generalizability may be limited. Therefore, future research could expand the sample size and scope of the study population to enhance its external validity. In addition, the cross-sectional study design limits inferences of causality.

Chinese female university students exhibited a high rate of HPV vaccine hesitancy (44.75%), with field of study, self-acquisition of HPV vaccine-related knowledge, and vaccine hesitancy identified as factors influencing students’ willingness to receive the HPV vaccine. Therefore, healthcare providers and educators should pay attention to the vaccine attitudes of female university students, reduce vaccine hesitancy, and enhance community education on cervical cancer, HPV infection, and prevention through effective multimedia campaigns. The aim of this approach is to address students’ concerns regarding the safety and efficacy of vaccines and increase their trust in vaccines, and this approach holds significant importance in promoting willingness to receive the HPV vaccine and improving vaccine coverage rates among female university students.

## Data Availability

The datasets used and/or analysed during the current study available from the corresponding author on reasonable request.
